# Protective effects of polysaccharides and polyhydric alcohols in a dry mouth model in cultured cells

**DOI:** 10.1007/s00520-011-1135-7

**Published:** 2011-04-09

**Authors:** Akiko Morito, Koichi Fujisawa, Toru Eguchi, Yojiro Ota

**Affiliations:** 1 Research and Development Department, Sunstar Inc., 5-30-1 Kamihamuro, Takatsuki, Osaka, Japan; 2 Research and Development Department, Sunstar Suisse SA, Etoy, Switzerland; 3 Dental and Oral Surgery Division, Shizuoka Cancer Center Hospital, Shizuoka, Japan

**Keywords:** Dry mouth, Oral mucosa, Cell assay, Xanthan gum, Gellan gum, Glycosyltrehalose

## Abstract

**Purpose:**

The goal of the study was to investigate the effects of 21 polysaccharides and 12 polyhydric alcohols on inhibition of dryness in oral mucosal epithelial cells in vitro. All the tested compounds are currently used in oral products.

**Methods:**

Human gingival epithelial Ca9-22 cells were incubated in 96-well plates until the cells reached confluence. After removal of the culture medium, the cells were incubated with a solution containing one of 21 polysaccharides (seven semisynthetic and 14 natural polysaccharides) or 12 polyhydric alcohols for 15 min (short-term treatment). After removal of the sample solution, the cells were dried at 25°C and 30% humidity, and cell viability was measured to determine the effect of each compound on prevention of cell death due to drying. The effects of the polyhydric alcohols were also examined for 3 days (long-term treatment).

**Results:**

The semisynthetic polysaccharides ethylcellulose (EC), methylcellulose (MC), and hydroxypropylmethylcellulose (HPMC) and the natural polysaccharides xanthan and gellan gum significantly inhibited cell death due to drying. Hydroxypropylcellulose increased cell death under these conditions. Of the polyhydric alcohols, long-term treatment with glycosyltrehalose significantly inhibited cell death due to drying, but short-term treatment with glycosyltrehalose did not do so. Long-term treatment had an effect on cell proliferation that appeared to differ from the effect of short-term treatment.

**Conclusions:**

Short-term treatment with EC, MC, HPMC, xanthan gum, and gellan gum and long-term treatment with glycosyltrehalose showed significant inhibition of cell death due to drying. These materials might have protective effects against dry mouth.

## Introduction

Healthy oral mucosa is covered with fluent mucous to protect the mucosa, epithelium, and teeth as a physical barrier from external stress; to reduce friction in the oral cavity and facilitate movement of the lips, tongue, cheek, and jaw; and to help articulation, conversation, swallowing, mastication, and taste perception [1, 2]. The mucous is produced from saliva, and a decrease in the amount of saliva disturbs these effects and results in a reduced quality of life (QOL). Dry mouth is a frequent symptom in the oral cavity of cancer patients undergoing treatment such as chemotherapy and radiotherapy in the head and neck region. These therapies induce various oral side effects and complications, including mucositis, dysphagia, infection, sensitivity, and dry mouth [3, 4]. The oral flora is changed in patients with dry mouth due to administration of drugs with adverse reactions [5] and radiotherapy in head and neck [6]. The change in oral flora can be caused by alternation of innate antimicrobial properties with decreased saliva. Human saliva contains a number of agents that protect oral tissues against noxious compounds produced by various microorganisms. The properties of salivary components which play innate defense roles change in people with poor saliva [5, 7–9]. Volatile sulfer compounds (VSC) are responsible for halitosis [10], and the risk of oral candidiasis and caries increases as the salivary secretion decreases [11]. In addition, dry mouth can change the oral mucosa and lead to a dried mucosal surface, mucosal flare, coated tongue, and atrophy of the tongue papillae. Such symptoms are caused by reduced self-protection by saliva [12]. For these reasons, severe dry mouth observed in oral cancer patients requires treatment with moisturizers.

Moisturizers for treatment of oral dryness are classified into agents for improving salivation and as saliva substitutes for retaining moisture in the oral cavity. Saliva-promoting agents include muscarinic agonists such as cevimeline hydrochloride and pilocarpine hydrochloride, which improve salivation through effects on the autonomic nervous system. Consequently, these drugs are associated with adverse reactions that reduce QOL, such as sweating, and may also cause gastrointestinal dysfunction [13–16]. In contrast, saliva substitutes have few adverse reactions. These agents are composed of polymer thickeners and polyhydric alcohols that retain moisture in the oral mucosa. However, most studies of these products have used questionnaires or rheological assessment in vitro to evaluate efficacy, neither of which is appropriate for objective assessment of their effects in humans [17–22]. In this study, the effects of these agents were evaluated in cell culture, which provides a better evaluation of the probable effect in vivo.

Few studies on evaluating the efficacy of dry mouth with cultured cell in vivo have been reported, and then we referred to the experiment on dry eye. The studies on the disorder of ocular mucosal cells found in experimentally induced dry eye have been reported [23, 24]. An evaluating system focused on the breakdown of epithelial cells in the conjunctiva and cornea, the surface layers of the eye, has been developed in cultured cells [25]. Matsuo showed that treatment of cells with 200 mM trehalose for 15 min after removal of culture medium prevented a subsequent drying-induced decrease in the viability of human corneal epithelial cells in vitro [26]. We adapted this approach to a system using oral gingival epithelial cells to examine the effects of polysaccharides and polyhydric alcohols on cell death caused by drying. In this study, we examined the inhibitory effects on dryness of short-term (15 min) and long-term (3 days) treatment with polysaccharides or polyhydric alcohols, based on the assumption that these compounds might be used clinically for single and continuous treatment.

## Materials and methods

### Cells and culture

Human gingival squamous carcinoma-derived Ca9-22 cells (Health Science Research Resources Bank, Tokyo, Japan) were incubated with Eagle's Minimum Essential Medium (Sigma, St. Louis, MO, USA) including 10% fetal bovine serum (Moregate Biotech, Bulimba, QLD, Australia) and 1% antibiotics (Antibiotic-Antimycotic: including penicillin G sodium, streptomycin sulfate, and amphotericin; Life Technologies Co., Carlsbad, CA, USA) at 37°C in an incubator with 5% CO_2_.

### Evaluation of inhibition of cell death caused by drying

Cells in the logarithmic growth phase were plated on 96-well plates (Sumitomo Bakelite, Tokyo, Japan) to reach confluence after 2 days (for short-term treatment) or after 4 days (for long-term treatment). Polysaccharides (Table 1) were dissolved in phosphate-buffered saline (PBS) to make a 0.02% solution. Polyhydric alcohols (Table 2) were diluted with PBS and medium to prepare 100 and 200 mM solutions for short- and long-term treatment, respectively. For long-term treatment, sample solutions were sterilized using a 0.22-μm filter (Millipore, Billerica, MA, USA).

**Table 1:** Polysaccharides used in the study

Polysaccharides	Supplier
Semisynthetic	
Ethylcellulose (EC)	Hercules, USA
Methylcellulose (MC)	Shin-Etsu Chemical Co., Ltd., Japan
Hydroxypropylcellulose (HPC)	Nippon Soda, Co., Ltd., Japan
Hydroxypropylmethylcellulose (HPMC)	Shin-Etsu Chemical Co., Ltd., Japan
Carboxymethylcellulose (CMC)	Daicel Chemical Industries, Ltd., Japan
Hydroxyethylcellulose (HEC)	Daicel Chemical Industries, Ltd., Japan
Cationized HEC	Toho Chemical Industry Co., Ltd., Japan
Natural	
Sodium alginate	Kibun Foodchemifa Co., Ltd., Japan
Sodium hyaluronate	Seikagaku Corporation, Japan
Sodium chondroitin sulfate	Nacalai Tesque, Inc., Japan
Xanthan gum	CP Kelco, USA
Carrageenan	CP Kelco, USA
Pullulan	Hayashibara Biochemical Laboratories, Inc., Japan
Gellan gum (deacylated type)	CP Kelco, USA
Locust bean gum F	San-Ei Gen F.F.I., Inc., Japan
Guar gum	Kyokuto Kagaku Sangyou Co., Ltd., Japan
Cationized guar gum	Toho Chemical Industry Co., Ltd., Japan
Cationized fenugreek gum	Toho Chemical Industry Co., Ltd., Japan
Mucin (Porcine gastric mucin)	Ichimaru Pharcos Co., Ltd., Japan
Glucomannan	San-Ei Gen F.F.I., Inc., Japan
Polysaccharides from tamarind seeds	San-Ei Gen F.F.I., Inc., Japan

**Table 2:** Polyhydric alcohols used in the study

Polyhydric alcohols	Supplier
Glycerin	Sakamoto Yakuhin Kogyo Co., Ltd., Japan
Propylene glycol (PG)	Mitsui Chemical Polyurethane, Inc. Japan
1,3-Butylene glycol (1,3-BG)	Daicel Chemical Industries, Ltd., Japan
Polyethylene glycol (PEG)	Dai-Ichi Kogyo Seiyaku Co., Ltd., Japan
Sorbitol	Towa Kasei Co., Ltd., Japan
Maltitol	Towa Kasei Co., Ltd., Japan
Xylitol	Nikken Chemical and Synthetic Industry Co., Ltd., Japan
Erythritol	B Food Science Co., Ltd., Japan
Palatinit	Mitsui Sugar Co., Ltd., Japan
Trehalose	Hayashibara Biochemical Laboratories, Inc., Japan
Glycosyltrehalose	Hayashibara Biochemical Laboratories, Inc., Japan

For short-term treatment, the medium was removed from the wells, the cells were washed with PBS, and the solution was removed. A sample solution was put in a well and incubated at 37°C for 15 min before being removed from the well. Wells with non-dried control cells were filled with 200 μL of PBS and sealed.

For long-term treatment, the sample was add to cell culture medium at a final concentration of 10 mM 1 day after plating and incubated for 3 days. After sample treatment, the medium containing the sample was removed and the wells were washed with PBS. Wells of the non-dried groups were filled with 200 μL of PBS and sealed (control group). Solution was removed from the wells of all dried groups.

In both treatments, cells were dried at 25°C and 30% humidity in a constant temperature and humidity chamber (Sanyo Electric Co., Osaka, Japan). After drying for 6 to 8 min, 200 μL of PBS was added to the wells of the plate.

### Determination of cell viability

Cell viability was measured using a reagent for counting viable cells, alamarBlue™ (AbD Serotec, Oxford, UK). Fluorescence from viable cells was measured at 560 and 590 nm as the absorption and emission wavelengths using a microplate spectrofluorometer (Gemini XPS; Molecular Devices, Sunnyvale, CA, USA). PBS-treated and non-dried cells were used as controls and the viability of these cells was set at 1. The viability of dried cells was then calculated as: Cell viability = (Fluorescence level of the treated group/Fluorescence level of the control group)

### Statistical analysis

For evaluation of short-term treatment, six plates for which the cell viability of the PBS-treated group after drying ranged from 0.2 to 0.5 were selected for analysis. Data for all samples were analyzed by one-way ANOVA and a post hoc Dunnett T3 test. For evaluation of long-term treatment, 13 plates for which the cell viability of the PBS-treated group after drying ranged from 0.1 to 0.5 were selected for analysis. Data for all samples were analyzed by one-way ANOVA and post hoc Tukey test. A two-tailed *P* value ≤0.05 was considered to indicate a significant difference in all analyses. Data were analyzed using SPSS Ver.13 (SPSS Inc, Chicago, IL, USA).

## Results

### Short-term treatment with polysaccharides

**Fig. 1:**
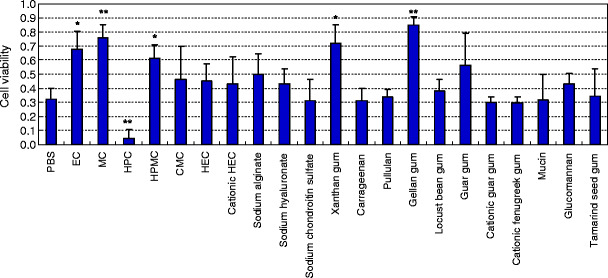
Cell viability after 15-minute treatment with polysaccharides followed by drying. Cells were incubated with each polysaccharide in a 0.02% solution in PBS and then dried after removal of the sample solution. The mean cell viability is shown relative to a cell viability of 1 for PBS-treated and non-dried (control) cells. The *error bars* indicate standard deviations. ANOVA and post hoc Dunnett T3 test: *p < 0.05 and **p < 0.01 vs. PBS

The inhibitory effect of polysaccharides on cell death caused by drying was evaluated in cultured human gingival epithelial Ca9-22 cells. The viability (mean ± standard deviation) of cells treated with polysaccharide solutions for 15 min after removal of the culture medium and then dried is shown in Fig. 1. The cell viability following treatment with PBS alone was 0.32; those with the semisynthetic polysaccharides ethylcellulose (EC), methylcellulose (MC), and hydroxypropylmethylcellulose (HPMC) were 0.68, 0.76, and 0.61, respectively; and those with the natural polysaccharides xanthan gum and gellan gum were 0.72 and 0.85, respectively. The viability was significantly higher with EC, HPMC, and xanthan gum (*p* < 0.05) and MC and gellan gum (*p* < 0.01) compared with PBS. The viability after hydroxypropylcellulose (HPC) treatment was 0.04, implying that HPC enhanced cell death due to drying.

### Short-term treatment with polyhydric alcohols

**Fig. 2:**
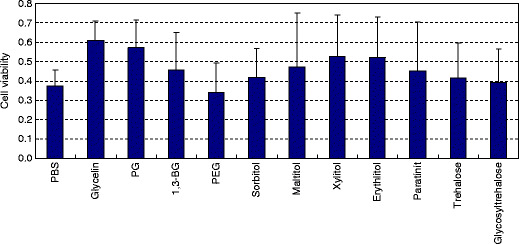
Cell viability after 15-minute treatment with polyhydric alcohols followed by drying. Cells were incubated with each polyhydric alcohol prepared at 100 mM in PBS for 15 minutes and then dried after removal of the sample solution. The mean cell viability is shown relative to a cell viability of 1 for PBS-treated and non-dried (control) cells. The *error bars* indicate standard deviations

The mean viabilities of cell dried after treatment with polyhydric alcohol solutions for 15 min are shown in Fig. 2. The viabilities following treatment with glycerin and PG were 0.61 and 0.57, respectively, which were more than 1.5-fold higher than that of 0.37 with PBS. However, the results of one-way ANOVA for the 12 polyhydric alcohols showed no significant differences.

### Long-term treatment with polyhydric alcohols

**Fig. 3:**
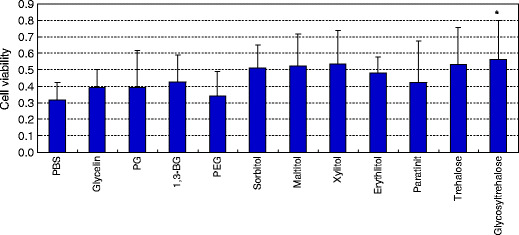
Cell viability after 3-day treatment with polyhydric alcohols followed by drying. Polyhydric alcohols at 10 mM in PBS were added to the cells one day after plating and incubated for 3 days. After removal of the medium and sample solution, the cells were washed with PBS and dried. The mean cell viability is shown relative to a cell viability of 1 for PBS-treated and non-dried (control) cells. The *error bars* indicate standard deviations. ANOVA and post hoc Tukey test: *p < 0.05 vs. PBS

Polyhydric alcohols were added to the cells 1 day after plating at 10 mM and the cells were incubated for 3 days. After removal of the medium and sample solution, the cells were washed with PBS and dried. The viabilities of the treated cells are shown in Fig. 3. The mean cell viability after treatment with glycosyltrehalose was 0.56, which was significantly higher than the cell viability of 0.32 with PBS alone (*p* < 0.05). The cell viabilities in long-term treatment with trehalose and glycosyltrehalose were higher than the respective values in short-term treatment.

## Discussion

The inhibitory effects of compounds on cell death due to dryness were screened in cultured oral epithelial cells in vitro. Human gingival squamous carcinoma-derived Ca9-22 cell was cultured conventionally in vitro, which validated capability to the test against drying with this cell as well as corneal epithelial cell. This screening approach identified several semisynthetic (EC, MC, and HPMC) and natural (xanthan gum and gellan gum) polysaccharides as agents that protect cells against damage due to drying. Short-term treatment with these compounds significantly improved cell viability and inhibited cell death compared to treatment with PBS alone. Semisynthetic polysaccharides with glucose units with low molecular weight methyl, ethyl, and hydroxypropyl groups were particularly effective. However, HPC, which contains only hydroxypropyl groups, was much less effective than HPMC. The cause of this result is unclear. The cell viability immediately after treatment for 15 min with HPC and without drying was not lower than that with PBS and other compounds (data not shown), which suggests that HPC promotes cell death under dry conditions.

Of the natural polysaccharides, xanthan gum, and gellan gum had strong protective effects against cell death caused by drying. Xanthan gum is a natural gum secreted by Xanthomonas bacteria. Xanthan gum is very thick, even at a low concentration, and consequently is widely used in cosmetics and foods. Xanthan gum shows a high bioadhesive effect in desquamated human buccal epithelial cells [27], and this adhesion might be related to the strong protective effect in gingival epithelial cells. Gellan gum is produced by Sphingomonas bacteria and is also used in various foods. These two compounds are anionic polymers. However, sodium alginate, sodium hyaluronate, sodium chondroitin sulfate, carrageenan, and mucin, which are also anionic polymers, had similar effects to nonionic guar gum, glucomannan, locust bean gum, polysaccharides from tamarind seeds, and pullulan. Therefore, not all anionic polymers had a strong protective effect. Screening of cationized guar gum, cationized fenugreek gum, and cationized HEC was also performed, but strong effects were not found. In addition, nonionic polymers had strong effects of the semisynthetic polysaccharides. Therefore, it is difficult to link the structural characteristics of the polysaccharides to their protective effects against cell death caused by drying. In short-term treatment, the most effective compounds were the semisynthetic polysaccharides EC, MC, and HPMC, and the natural polysaccharides xanthan gum and gellan gum. Because the two natural polysaccharides are used in food and are safe for swallowing, they may be preferable to semisynthetic polysaccharides for use in oral products for people with reduced physical strength, such as cancer patients.

The polyhydric alcohols screened in the study had low viscosity and can be treated with sterile filtration. Therefore, in addition to evaluation of their effect after treatment for 15 min, the effect of long-term treatment for 3 days, in which the cells reached confluence 1 day after plating, was also evaluated. With polyhydric alcohols, the cell viability after drying in the short-term treatment was approximately 0.6 for glycerin, which was higher but not significantly different to that with PBS. However, in long-term treatment, glycosyltrehalose gave significantly higher cell viability than PBS, although this viability was lower than in short-term treatment. Glycerin and PG had relatively strong effects in short-term treatment, but did not show protective effects that differed significantly from PBS in long-term treatment. The increased cell viability with long-term treatment with trehalose and glycosyltrehalose suggests that trehalose may change the cellular structure to produce resistance to damage by drying. In this study, we tested glycosyltrehalose, which is mixture of trehalose derivatives and is mainly maltosyltrehalose. Based on information provided by the supplier, glycosyltrehalose has protective effects against ultraviolet radiation in human fibroblasts and against sodium dodecyl sulfate stimulation in human skin tissue. These effects of glycosyltrehalose on external stresses may have led to the results in this study.

Matsuo [26] showed that trehalose suppressed the reduction in cell viability of cultured human corneal epithelial cells caused by drying. It has also been suggested that trehalose improves disorders due to drying through suppression of apoptosis in ocular surface epithelial cells [28], and trehalose solution is a better treatment for moderate to severe dry eye syndrome for 4 weeks in comparison with two uncompounded eye drops [29]. Long-term treatment with glycosyltrehalose had similar protective effects on human gingival epithelial cells in this study, which suggests that trehalose derivatives can increase cellular resistance to drying regardless of the cell strain.

We examined the protective effects of short-term and long-term treatment on the assumption that the compounds might be used clinically for both single and continuous treatment. Severe dry mouth requires quick-acting relief through a single treatment and also continuous daily care. In the respective methods used in this study, different materials showed significant protective effects, which suggests that there are different mechanisms of protection against cell death caused by dryness. The results also indicate that products with a combination of the materials found to be effective in short-term and long-term treatment might have greater preventive effects against oral dryness. Finally, we note that the compounds were examined using a monolayer cell culture system with the assumption that this will reflect their effects in vivo. For practical use of products containing the effective materials, clinical evaluations are required in patients with dry mouth, such as that due to oral cancer.

## Conclusion

An in vitro evaluation system using cultured human oral mucosal epithelial cell line was used to determine the inhibitory effects of polysaccharides and polyhydric alcohols on cell death caused by drying. Cell viability in comparison with that of PBS-treated cells was used as an indicator. The results showed that the semisynthetic polysaccharides EC, MC, and HPMC; the natural polysaccharides xanthan gum and gellan gum; and the polyhydric alcohol glycosyltrehalose had significant protective effects against dryness.
